# Notes and News

**Published:** 1895-01-19

**Authors:** 


### Notes and News.

Collected and Communicated.

At Turin a sanitary congress is to take place in September next.

The Council of the Metropolitan Hospital Saturday Fund will distribute £17,000 amongst the medical charities of London.

The Queen has been pleased to accept a copy of "Infant Feeding by Artificial Means: A Scientific and Practical Treatise on the Dietetics of Infancy," by Mrs. S. H. Sadler.

Those who must of necessity dwell in the metropolis should feel encouraged by hearing that at this inclement time of year the health of London is better than in most provincial towns. The death-rate lately has been markedly below the average.

Lord Brassey has consented to take the chair at the annual court of the Seamen's Hospital Society, to be held on February 20th, at four o'clock, at Sion College, on the Victoria Embankment, and Mr. Leopold de Rothschild is announced to preside at the Annual Festival Dinner for the Metropolitan Hospital on June 13th.

Sir W. Broadbent, Sir William Priestley, Sir Walter Foster, and Mr. Henry Morris have been invited to assist the Board of Management of the Chelsea Hospital for Women in selecting a new medical staff. It is to be regretted that the profession did not earlier know of the important names to be included in the committee of selection.

Professor Burdon Sanderson has succeeded Sir Henry Acland as Regius Professor of Medicine at Oxford. The new professor has for some years held the post of Wynflete Professor of Physiology, and is one of the most eminent physiologists of the day. He is a leader in the advanced school of science, and is regarded as a thorn in the side of anti-vivisectionists, yet he took a prominent part in the experiments organised by the Humane Society to establish the best method of restoring animation to drowning persons.

A strange and severe epidemic has appeared in Samoa. Many Europeans have been attacked, and the native mortality is very large. Medical aid has been able to do very little to check the spread of the disease.

Although the Pasteur methods are by no means unchallenged, the great pioneer is not without honour in his own country. The Municipal Council of Paris are about to discard the name of the Rue d'Ulm, and call it henceforth Rue Pasteur.

We are glad to learn that Professor Herkomer is setting residents of Oxford and the county an example in the active interest he is taking in the Radcliffe Infirmary. Professor Herkomer is organising an exhibition of his works at Oxford, the proceeds of which are to go to the infirmary.

The memory of the late Tsar of Russia is to be honoured by the English residents at St. Petersburg. The memorial is to take the form of two endowed beds at the Women's Hospital of the Society of the Sisters of Mercy of the Holy Trinity. If it is a fact that the sum already collected amounts to £5,300, the memorial should take a much larger form than this.

The case of the alleged sham orphanages at Worthing and elsewhere, just at present occupying the attention of the Law Courts, should act as a further warning to the giving of charity without sufficient enquiry. The very simple expedient of referring to Burdett's "Hospitals and Charities Annual" would prevent many unfortunate mistakes, and lead funds into worthy channels.

Amongst the many new periodicals which have appeared at the beginning of the present year, we would especially draw attention to the contribution to medical literature under the title of "Clinical Sketches." This is a new monthly magazine, devoted to subjects which are of interest to the general practitioner, and others interested in medical science. The first number is an excellent specimen. The contents consist of original clinical lectures or papers, short notes of cases, abstracts and *résumés* of current literature, clinical notes, and descriptions of new apparatus. And later, we learn, that reviews of new books and new editions will be added to this list. We think the magazine fills a distinct want, and we wish it every success.

A public meeting, in aid of St. Thomas's Hospital, is to be held at the Mansion House on Wednesday, February 13th, at three o'clock, when the Lord Mayor will preside, supported by H.R.H. the Duke of Connaught, president of the hospital. On this occasion an appeal for £100,000 will be made towards the opening of the wards of the hospital which are at present closed. Since 1874 the in-patients have increased from 3,500 to 5,940 per annum, the out-patients having nearly doubled their numbers during the same period. The hospital is entirely free, no letters of admission being allotted to contributors. St. Thomas's, and one other hospital, are the only two institutions of the kind in South London. More accommodation for the sick poor is urgently needed. This, however, cannot be provided unless further funds are forthcoming.

